# Nanoparticle-enhanced PD-1/PD-L1 targeted combination therapy for triple negative breast cancer

**DOI:** 10.3389/fonc.2024.1393492

**Published:** 2024-05-02

**Authors:** Caroline Linde, Yu-Ting Chien, Zhiqian Chen, Qingxin Mu

**Affiliations:** Department of Pharmaceutics, University of Washington, Seattle, WA, United States

**Keywords:** nanoparticles, triple negative breast cancer, PD-1/PD-L1 pathway, combination therapy, tumor microenvironment

## Abstract

Breast cancer with triple-negative subtype (TNBC) presents significant challenges with limited treatment options and a poorer prognosis than others. While PD-1/PD-L1 checkpoint inhibitors have shown promise, their efficacy in TNBC remains constrained. In recent years, nanoparticle (NP) technologies offer a novel approach to enhance cancer therapy by optimizing the tumor microenvironment and augmenting chemo- and immunotherapy effects in various preclinical and clinical settings. This review discusses recent investigations in NP strategies for improving PD-1/PD-L1 blockade-based combination therapy for TNBC. Those include single or multi-therapeutic NPs designed to enhance immunogenicity of the tumor, induce immunogenic cell death, and target immunosuppressive elements within the tumor microenvironment. The investigations also include NPs co-loaded with PD-L1 inhibitors and other therapeutic agents, leveraging targeted delivery and synergistic effects to maximize efficacy while minimizing systemic toxicity. Overall, NP approaches represent a promising avenue for enhancing PD-1/PD-L1 checkpoint blockade-based combination therapy in TNBC and encourage further developmental studies.

## Introduction

Breast cancer is the second leading cause of cancer death in women worldwide (1). The subtype that lacks ER/PR and HER2 receptors (triple negative breast cancer, TNBC) have 5-year survival rates that are 8-16% lower than hormone-positive subtypes (2). Current treatments mostly involve surgical removal of primary tumor with chemotherapy and radiation (3). Monoclonal antibodies targeting PD-1/PD-L1 pathway (i.e., pembrolizumab) have been recently added as a first line treatment with chemotherapy due to their longer overall survival than chemotherapy alone (23 months vs. 16.1 months) (4). However, efficacy of these regimens relies on tumoral PD-L1 expression (combined positive score [CPS] ≥10) (4). Despite improved survival, the combination regimens still showed adverse side effects (4), and their synergistic effects might not be optimal due to their different pharmacokinetic profiles (5). Another PD-1/PD-L1 inhibitor atezolizumab initially showed an improvement in progression-free survival regardless of PD-L1 expression (7.2 months vs. 5.5 months) when combined with the NP nab-paclitaxel, but did not show any benefit (6 months vs. 5.7 months) when combined with free paclitaxel, even for PD-L1 positive patients according to the IMpassion131 trial (6). Therefore, innovative approaches to further improve treatment outcomes are still highly demanded for TNBC (6).

Nanoparticle (NP) technologies have been investigated to improve the outcomes for various cancers including TNBC. NPs can load single or multiple agents and deliver them in controlled and targeted manner, thus lessening the toxicity and pharmacokinetic issues of free drugs (7, 8). They are proven to be useful when combining immune checkpoint blockade with drugs that stimulate the immune system (e.g., through immunogenic cell death mechanisms) (9), and thus would be more effective than free agents to combat the immunosuppressive TNBC tumor microenvironment and enhance the PD-1/PD-L1 blocking therapy. One example is the FDA-approved albumin-bound paclitaxel formulation known as nab-paclitaxel, a ~130 nm NP formulation (10), which shows faster drug distribution into tissues and slower elimination than solvent- and surfactant-based Taxol in clinical trials (11). The NPs improved the treatment outcome of anti-PD-1 antibody pembrolizumab for the treatment of TNBC as mentioned above (4, 12).

**Figure 1:**
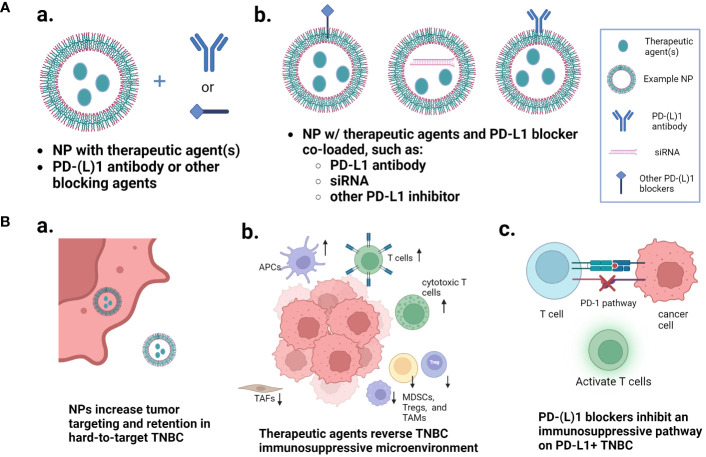
Nanoparticle approaches that improve the PD-1/PD-L1 pathway inhibition-based combination therapy of TNBC. **(A)** Two major types of nanoparticle loading and combination approaches: nanoparticles loaded with one or more therapeutic agents and combined with free PD-1/PD-L1 blocker separately, or nanoparticles co-loaded with the immune checkpoint inhibitor and other therapeutic agents. **(B)** Multiple mechanisms that are involved in the enhanced therapeutic outcomes. (a) multi-agent nanoparticles increase the tumor targeting and retention in hard-to-target TNBC; (b) the therapeutic agents reverse the TNBC immunosuppressive microenvironment; and (c) the PD-1/PD-L1 blockers inhibit the immunosuppressive pathway. Figure created with BioRender.com.

In this mini review, we will discuss recent innovative developments in the use of NP technologies to improve the therapeutic outcomes of PD-1/PD-L1 therapy of TNBC. First, we will give a brief introduction of the PD-1/PD-L1 signaling axis and an overview of the clinical studies that have investigated the immune microenvironment of tumors before they are treated with anti-PD-1/PD-L1 therapy combined with nab-paclitaxel or liposomal doxorubicin. Then we will discuss the investigations of preclinical NPs that are either loaded with single or multiple therapies to optimize the tumor microenvironment for combination with separately administered PD-1/PD-L1 blocking therapy, or NPs co-loaded with the immune checkpoint blocking agents and other therapies (**Figure 1**). The detailed information of these NPs, including NP types, preparation methods, therapeutic agents, therapeutic efficacy with immunological evaluation, and safety/toxicity assessments, are summarized in **Supplementary Table S1**.

## PD-1/PD-L1 axis in combination therapy of TNBC

The binding of cancer cell surface PD-L1 (programmed death ligand 1) to PD-1 (programmed death receptor 1) on T cell surface causes the termination of effector T cell proliferation and subsequent apoptosis, resulting in cancer cell survival (13, 14). Expression of PD-L1 in TNBC has been correlated with both positive and negative clinical prognoses, according to different studies. Some have noted that it is associated with a large tumor size and lymph node metastasis, while others have claimed that it is associated with higher tumor infiltrating lymphocytes and shows a good clinical outcome (13). This could be due to different methods of identifying the PD-L1 expression level, the number of tumor infiltrating lymphocytes found in the tumor microenvironment, and whether PD-L1 is found on the primary tumor or on metastatic cells (14). Anti-PD-1 or anti-PD-L1 blocking antibodies for TNBC must be used with chemotherapy for neoadjuvant treatment (15). Chemotherapeutics may have immunomodulative effects through multiple mechanisms including immunogenic cell death (ICD), enhanced antigen presentation and MHC-I molecules, dendritic cells activation, and decreased myeloid derived suppressor cell (MDSC) levels, etc. (16). Some of them upregulate PD-L1 protein expression in tumors, which enhances PD-L1 antibody therapy (17). However, most of these drugs are non-cancer specific and have short circulation, thus resulting in suboptimal outcomes when combined with the PD-1/PD-L1 antibodies. Phototherapy (photothermal or photodynamic) is another modality that can induce ICD (18), and tumor normalization strategies can allow for improved immune cell infiltration, thus improving immunotherapy (19). In this mini review, different NP approaches are used to improve the effects of different therapeutics in optimizing the tumor microenvironment for PD-1/PD-L1-targeted therapy.

## Clinical studies of nanoparticle formulation-PD-1/PD-L1 inhibitor combination therapy

Increased immune activity in the tumor before treatment is correlated with the efficacy of nab-paclitaxel and pegylated liposomal doxorubicin when combined with anti-PD-1/PD-L1 therapy (**Table 1**). A clinical study that combined nab-paclitaxel with pembrolizumab showed that a complete response was correlated with increased immune activity in the pre-treated tumor through a gene set enrichment analysis (20). Another study that combined nab-paclitaxel with atezolizumab showed that tumor infiltrating lymphocytes were associated with PD-L1 positive tumors, both of which were correlated with progression-free survival and overall survival (21). A study combined atezolizumab with pegylated liposomal doxorubicin and cyclophosphamide and showed that high immune gene expression in the tumor seemed to be essential for atezolizumab to be effective (22). These studies demonstrate that increased immune activity in the pretreated tumor microenvironment can lead to increased efficacy when nanoparticles are combined with anti-PD-1/PD-L1 therapy. It’s worth noting that these nanoformulations are intravenously infused, which provides a 100% bioavailability to the systemic circulation.

**Table 1:** Examples of nanoparticle formulations used in combination with PD-(L)1 inhibitors for TNBC treatment in the clinic.

Regimen	Primary Outcomes	Immunological Assessments	Ref.#
Pembrolizumab + carboplatin, nab-paclitaxel	• PFS*: 5.8 months • OS*: 13.4 months • ORR*: 48%	• Tumors from patients w/CR* had increased gene set enrichment for: B cell receptor antigen activation, PD-1 signaling, TCR signaling, MHC Class II antigen presentation, and antigen processing-cross presentation • IGHG1* enriched expression correlated with higher B cell and helper T cell tumor infiltration and led to ~60% probability of 20–40-month OS and >75% probability of PFS from 10-40 months	(20)
Atezolizumab + nab-paclitaxel	• PFS: 9.3 months in PD-L1 IC*+ patients • OS: 28.9 months in PD-L1 IC* + patients	• Intratumoral CD8+ and sTIL+* were associated with PD-L1 IC+ status and improved PFS and OS	(21)
Arm 1: PLD* + cyclophosphamide + atezolizumab Arm 2: PLD + cyclophosphamide+ Placebo	Arm 1: • PFS: 4.3 months • ORR: 27.5% Arm 2: • PFS: 3.5 months • ORR: 17.9%	• Only patients with high immune gene expression in the tumor seemed to benefit from the addition of atezolizumab • Patients with high immune gene expression all had a PFS >12 months • Low Treg levels were associated with increased PFS	(22)

*CR, Complete Response; IGHG1, Immunoglobulin Heavy Constant Gamma 1; sTIL, Stromal tumor infiltrating lymphocytes; IC, immune cell; PLD, pegylated liposomal doxorubicin; PFS, Progression Free Survival; OS, Overall Survival; ORR, Overall Response Rate.

## Single or multi-therapeutic preclinical nanoparticles that combine with anti-PD-1/PD-L1 therapy

Some preclinical studies utilized NPs to carry a single novel therapeutic function (similar to nab-paclitaxel) to increase the immunogenicity of the tumor. Wang et al. encapsulated the chemotherapeutic drug camptothecin in a liposome (Camptothesome). They combined alpha-PD-L1 (αPD-L1) with Camptothesome for treatment of 4T1-Luc2 tumors. The NP is internalized by clathrin-mediated endocytosis and escapes efflux pumps that confer multidrug resistance. In comparison with Onivyde (FDA-approved irinotecan liposomes), Camptothesome was able to upregulate PD-L1 levels by about 2.5-fold in addition to inducing ICD. When combined with αPD-L1, camptothesome reduced the 4T1 tumor volume by 80-90% compared to vehicle control (23). Liu et al. encapsulated the Hsp90 inhibitor 7-AAG in a liposome, which increased ICD rather than cytotoxicity. They also used an aminoethyl anisamide ligand to target the tumor. With this NP, the tumor infiltrating T cell population in 4T1 tumors increased by 10× and the memory CD8+ T cell population increased to about 7% versus the 1% in the vehicle control. When combined with αPD-L1, the tumor mass was reduced by ~50% (19). In another study by Zhao et al., black phosphorous quantum dots were coated with a cancer cell membrane for photothermal therapy of 4T1 tumor using an 808 nm laser irradiation. The cancer cell membrane increased NPs’ stability through reducing immune system clearance, and it also increased tumor-site accumulation. The release of tumor associated antigens induced by photothermal therapy increased the dendritic cell population in tumors to 32.80% versus 9.96% in the vehicle control. When combined with αPD-L1 and near infrared light (NIR), nearly all tumor mass was eliminated and the survival of the mice was still 100% after 2 months, whereas it was 0% for αPD-L1 only group and the PBS control (18). Another study targeted tumor associated fibroblasts (TAFs) by reducing ROS levels, utilizing the traditional medicine puerarin carried in a nanoemulsion (24). TAFs are an intertumoral cell population that releases growth factors, recruits immunosuppressive cells, and helps form a dense extracellular matrix that blocks drug delivery (25). The nanoemulsion also included a aminoethyl anisamide targeting ligand. In a 4T1 mouse tumor model, their NPs reduced alpha-SMA positive TAFs to 3.5% of total cells, and reduced collagen deposition (indicative of desmoplasia) in cells to 2.6% of total cells. When combined with αPD-L1, the tumor mass was decreased by about half, and the median survival time was 56 days, versus 41 days for NanoPue treatment only, and 38 days for αPD-L1 treatment only (24). It was also noted that all these NP-based regimens inhibited lung metastasis.

NPs were also used to carry dual agents to increase tumor immunogenicity. Feng et al. formulated NPs that were self-assembled from indocyanine green and paclitaxel without using additional excipient. The photodynamic effects increased dendritic cell maturation frequency to ~50% in the 4T1 tumor, and paclitaxel reduced T regulatory cells by 10-15% through cytotoxic effects. Combined with αPD-L1, this NP nearly eradicated the primary tumor and prevented lung metastasis in the 4T1 tumor. 75% of the mice survived after 45 days, versus around 30% without αPD-L1, and none survived only with αPD-L1 (26). Another study by Lu et al. used photothermal therapy by developing a polyethylene-glycol modified polydopamine (a polymeric photothermal conversion agent) nano-construct, loaded with the toll-like receptor 7 agonist R848 and carbon dots (a biocompatible nanomaterial). R848 was designed to increase cytotoxic T lymphocyte infiltration: with αPD-L1, the NP treatment led to a 2.1% cytotoxic T lymphocyte population in tumor tissue versus 1% without αPD-L1, 0.6% without R848 and 0.2% without treatment. Their NP, combined with near-infrared light irradiation and αPD-L1, decreased 4T1 tumor mass in mice by 75% compared to untreated mice. Finally, 80% of mice survived after 30 days, while none survived without R848 or in the control group (27).

A couple of NP approaches carried three or more agents to form more intricate and sophisticated systems, both involving the ROS induction mechanism to induce ICD. This is in contrast to the puerarin nanoemulsion study that decreased ROS levels because chronic ROS can activate TAFs (24). Zou et al. combined doxorubicin, adjudin (a male contraceptive and a potential anti-metastasis agent), and D-α-tocopherol polyethylene glycol 1000 succinate (TPGS, an ROS inducer), into a self-assembled NP with cRGD modification. These NPs had a pH-sensitive Schiff base and could release their drugs when endocytosed in lysosomes with a pH of 5. They increased the ROS levels by 2.3-fold compared to free doxorubicin in 4T1 cells. The authors specifically used an anti-metastasis agent because chemotherapy can induce the epithelial-mesenchymal transition effect that promotes metastasis. This study also used a free D-peptide antagonist to block PD-L1 instead of the antibody, to avoid the antibody-associated immune adverse effects (28). Li et al. encapsulated epirubicin, Gox and hemin into zeolitic imidazolate framework NPs that degrade at low pH (about 10× more epirubicin was released in pH 5.4 than pH 7.4). They then coated the NPs with calreticulin-overexpressed cancer cell membranes. The NPs increased the ROS content by ~2.5 folds in 4T1 tumor tissue (23). These studies showed similar 4T1 tumor inhibition rate (84.08% and 82.02%, respectively) when combined with αPD-L1 without detectable metastasis in the lungs (28, 29).

## Nanoparticles co-loaded with PD-L1 inhibitor and other forms of therapy for TNBC

Co-loading a PD-L1 inhibitor and other therapies onto NPs allows for synergistic effects through co-delivery into the tumor and may help reduce immunological side effects. A micelle encapsulating camptothecin and JQ1 was studied by Zhang et al, combining the potent chemotherapy with JQ1 (a bromodomain and extraterminal domain inhibitor that suppresses PD-L1 expression), solving the issues of the agents’ poor solubility, short half-lives, and off-target toxicity. Both drugs are conjugated to the polymer backbone via disulfide bonds, susceptible to the high levels of glutathione present within cancer cells. This demonstrated an 80.3% 4T1 tumor inhibition rate compared to 54.0% in the free CPT and JQ1 group (30). A study by Zhang et al. combined Ce6 (a photosensitizer) with a novel PD-L1 blocker known as Bristol-Myer’s Squibb 202 (BMS-202) and formulated them into NPs using a reprecipitation method. Under near infrared light, this BMS 202/Ce6 NPs treatment showed similar efficacy to Ce6 NPs combined with separately administered αPD-L1 in inhibiting the 4T1 tumor (91.1% vs. 92.6%) (31).

A few recent studies involved the PD-L1 inhibitor metformin in their formulation. Metformin causes the degradation of PD-L1, circumventing the transient nature of PD-L1 blocking with antibodies (32). One study first conjugated Ce6 with metformin through an MMP-2 cleavable peptide. The conjugate then self-assembled into NPs. The NPs had led to a 20% reduction in PD-L1 expression on 4T1 tumor cells and reduced the tumor weight by nearly 61.5% (33). Another designed NP that was self-assembled from metformin and chemotherapy SN38. This NP reduced PD-L1 expression by ~4× in MDA-MB-231 cells at an 80 ug/mL concentration, decreased relative tumor volume by 80% in the 4T1 BALB/c mouse model and prevented lung metastasis. The survival rate was 50% after 30 days with this NP as opposed to 0% in the free drug and free αPD-L1 group (34). In another study, an immunomodulator epigallocatechin gallate palmitate (PEGCG) and metformin were self-assembled into micelles and then co-loaded with doxorubicin (PMD NPs). Epigallocatechin gallate induces apoptosis of MDSCs and binds to laminin receptors highly expressed on breast cancer cells; PMDs and separate administration of anti-PD-1 antibody reduced MDSCs by 10% compared to the PBS control. They showed a tumor inhibition rate of 68.8% when anti-PD-1 antibody was separately administered (35). In addressing the multifaceted nature of TNBC, some more ambitious and intricate nanomedicine designs were also noted. For example, Zhang et al. fabricated NPs composed of 4 agents to target cancer in multiple ways: paclitaxel, repertaxin (a cancer stem cell antagonist), BMS-1 (a PD-1/PD-L1 pathway blocker), and combretastatin A4 (targets tumor micro vessels). This nanomedicine had 100% drug loading capacity through self-assembly, obviating the need for additional carriers which reduced the risk of carrier-induced side effects. Moreover, such design allowed for the low dose of paclitaxel (2 mg/kg compared to 10 mg/kg in common dosing regimens), which minimized systemic toxicity and the killing of immunocytes. Through repertaxin, this NP reduced cancer stem cells to 4.84% on day 12 from 12.7% in the PBS group. The CA4 in the NPs reduced the tumor micro vessel density by half compared to the PBS control. With these advantages, this multi-agent approach led to significant tumor growth inhibition (92.5%) and lung metastasis suppression (>90%). 50% of the mice had their survival prolonged by 95 days (36). Another study used a heparanase-sensitive micelle approach to co-deliver docetaxel, NLG919, and a PD-1/PD-L1 inhibitor, HY19991. NLG919 is an inhibitor of indoleamine 2,3-dioxygenase, a protein that causes the death of CD8+ T cells and promotion of Treg cells. The design allows for a controlled release of the drugs in the tumor in response to heparanase and uses monocytes to phagocytize the micelles and deliver them into the tumor. The monocytes prevented the NPs from being eliminated and releasing drugs in the blood. This delivery approach resulted in high intratumoral drug concentrations of all three agents (6.85, 3.96, and 3.11 times higher for docetaxel, NLG919, and HY, respectively), when compared to the free micelles (no monocyte carrier) in a 4T1 tumor model. When compared to the free drugs, the intratumoral drug concentrations were 7, 4.7, and 4.5 times higher respectively at 8 hours. Such enhancement ultimately led to a tumor inhibition rate of over 90% and suppression of 4T1 lung metastasis by ~98% compared to saline control. The free micelle tumor inhibition rate was only 48.51%. Furthermore, after 60 days, 66.67% of the mice treated with the monocyte-carried NPs still had survived, whereas the median survival length of the mice that were treated with free drugs was only 30 days (37).

Some other studies used anti-PD-L1 antibody-conjugated NPs to deliver drugs into PD-L1 high expressing tumor cells. In the study of Pham et al., human serum albumin NPs were loaded with paclitaxel through a pH-sensitive linker and conjugated with anti-PD-L1 antibody. The design leveraged the glycoprotein60 receptor-binding effects of albumin, the PD-L1 targeting ability, and a pH-dependent release of paclitaxel and the PD-L1 antibody. This formulation was combined with separately administered CTLA-4 antibody for treatment evaluations. In an EMT-6 tumor xenograft model, the NPs combined with free anti-CTLA-4 were able to decrease tumor mass by ~80%, in contrast to ~60% decrease without CTLA-4 antibody, and ~40% with nab-paclitaxel only (38). In another study, an oleic acid conjugated polyethyleneimine polymer complex was formed and loaded with paclitaxel, chloroquine (an autophagy inhibitor), ovalbumin (antigen), CpG (immunoadjuvant), and anti-PD-L1 antibody, through a thin-film hydration and a self-assembly process. The design enabled targeted tumor and lymph node delivery and durable antitumor immunity. They showed that autophagosome formation was promoted over 4× compared to the blank nanoparticle control, and that in a 4T1 tumor model, the tumor inhibition rate was ~80%. After 60 days, 60% of the mice treated with this NP had survived, in contrast to 50% for mice treated with a NP with no PD-L1 antibody and 0% for the 5% glucose (D5W) control (39).

Finally, PD-L1 siRNA (siPD-L1) with high specificity to targeted sequences was also utilized recently with NP approaches. In one study, siPD-L1 was first combined with protamine to form a cationic nanocore. LY3200882, a TGF-beta inhibitor that deactivates TAFs, was then combined with a MMP2-responsive lipid layer as the liposomal out-shell. Enabled by the MMP2-stimulated disruption of the outer layer, this design facilitated the siPD-L1/protamine inner core’s penetration of both tumor cells and TAFs. The NP downregulated α-SMA by 31.5% and Collagen 1 by 52.4% in NIH/3T3 cells, which showed that it could target TAFs and thus normalize the tumor microenvironment. In a 4T1 and NIH/3T3 co-inoculated tumor model (for a highly desmoplastic microenvironment), their NPs decreased the tumor mass by about 85% (40). In another study, the NPs were prepared by first forming a complex between melittin and PD-L1 DsiRNA (Dicer-substrate siRNA) followed by the loading of doxorubicin. The surface of the particles was then modified with hyaluronic acid (HA), which binds to CD44 expressed on some TNBC cells and which can be degraded by hyaluronidase in the ECM (allowing for drug release). The HA coating resulted in a 3× higher uptake of drugs in 4T1 cells compared to NPs with no HA coating and free doxorubicin. The NPs also induced a 75% decrease in PD-L1 expression in 4T1 tumors as compared to the PBS control. This resulted in a substantial reduction in 4T1 tumor weight, by about 75-80%, with all the mice surviving after 40 days and no surviving mice in the free drug groups (41).

## Summary and outlook

Despite recent clinical advancements of PD-1/PD-L1 inhibitor-based chemo-immunotherapy for TNBC, the therapeutic benefits and applicable patient populations remain limited (4). Various drug discovery and delivery approaches are being investigated to further augment the immune checkpoint inhibitors’ efficacy either alone or in combination with other modalities. NPs, owing to their unique physicochemical properties and multifunctional capabilities, have been investigated for the enhanced therapy of TNBC and have demonstrated advantages in both clinical [e.g., nab-paclitaxel NPs (10, 11)] and preclinical settings. Based on recent developments, multiple mechanisms may have contributed to their enhanced therapeutic outcomes, those include but are not limited to 1) reduced drug resistance (23, 42), 2) improved drug solubility and pharmacokinetics (7, 8, 11), 3) active tumor targeting through ligand or specialized membrane coating, etc. (18, 19, 24, 29, 31, 33, 35–41), 4) NP-enabled co-delivery of agents for better synergistic effects (27–31, 33–41), and 5) stimuli-responsive drug release (28, 37, 38). With these mechanisms, the NPs not only led to increased cytotoxic effects to cancer cells, but also optimized the tumor microenvironment for immunotherapy. The combination therapy with PD-1/PD-L1 blocking agents, either co-delivered by NPs or separately administered were hence improved through enhanced efficacy and reduced systemic toxicity, and ultimately, prolonged survival. It was also noted that these NPs are generally considered safe as assessed in each study without any significant toxicity being observed (**Supplementary Table S1**). We also noted that most of these studies used PD-L1 other than PD-1 inhibitors, likely taking the advantages of NPs being able to target cancer cells rather than immune cells. These recent advancements with NP strategies demonstrated in preclinical murine cancer models manifest their advantages and potential in treating TNBC in humans. Despite these advantages, some challenges remain. For example, consistent size distribution, shape and structure, surface charge, and compositions are required for the NP formulations, which could complicate the manufacturing processes (43, 44). Also, the previously claimed EPR (enhanced permeability and retention) effect-based NPs have shown different results in rodent models and humans, indicating that the NPs need to be further optimized or re-designed to fit for the tumor pathophysiology in humans (7, 45). In light of all above advantages and challenge considerations, further formulation development and optimization in the manufacturing of NPs, evaluations of NPs’ pharmacokinetics and drug release mechanisms, and efficacy and safety assessments in multiple late-preclinical models (such as humanized tumor models), are encouraged before translating into clinical investigations.

## Author contributions

CL: Conceptualization, Writing – original draft, Writing – review & editing. Y-TC: Writing – original draft, Writing – review & editing. ZC: Writing – original draft, Writing – review & editing. QM: Conceptualization, Funding acquisition, Writing – original draft, Writing – review & editing.

## References

[1] Giaquinto AN Sung H Miller KD Kramer JL Newman LA Minihan A. Breast cancer statistics, 2022. CA: A Cancer J Clin. (2022) 72:524–41. doi: 10.3322/caac.21754 36190501

[2] Howard FM Olopade OI. Epidemiology of triple-negative breast cancer: A review. Cancer J. (2021) 27:8–16. doi: 10.1097/PPO.0000000000000500 33475288 PMC12050094

[3] Baranova A Krasnoselskyi M Starikov V Kartashov S Zhulkevych I. Triple-negative breast cancer: current treatment strategies and factors of negative prognosis. J Med Life. (2022) 15:153–61. doi: 10.25122/jml-2021-0108 PMC8999097 35419095

[4] Cortes J Rugo HS Cescon DW Im SA Yusof MM Gallardo C. Pembrolizumab plus chemotherapy in advanced triple-negative breast cancer. N Engl J Med. (2022) 387:217–26. doi: 10.1056/NEJMoa2202809 35857659

[5] Lee JH Nan A. Combination drug delivery approaches in metastatic breast cancer. J Drug Deliv. (2012) 2012:915375. doi: 10.1155/2012/915375 22619725 PMC3350970

[6] Nunes Filho P Albuquerque C Capella MP Debiasi M. Immune checkpoint inhibitors in breast cancer: A narrative review. Oncol Ther. (2023) 11:171–83. doi: 10.1007/s40487-023-00224-9 PMC10260715 36917399

[7] Wu J. The enhanced permeability and retention (EPR) effect: the significance of the concept and methods to enhance its application. J Pers Med. (2021) 11:771. doi: 10.3390/jpm11080771 34442415 PMC8402171

[8] Gabizon A Shmeeda H Barenholz Y. Pharmacokinetics of pegylated liposomal Doxorubicin: review of animal and human studies. Clin Pharmacokinet. (2003) 42:419–36. doi: 10.2165/00003088-200342050-00002 12739982

[9] Qi J Jin F Xu X Du Y. Combination cancer immunotherapy of nanoparticle-based immunogenic cell death inducers and immune checkpoint inhibitors. Int J Nanomed. (2021) p:1435–56. doi: 10.2147/IJN.S285999 PMC7910111 33654395

[10] Iglesias J. nab-Paclitaxel (Abraxane®): an albumin-bound cytotoxic exploiting natural delivery mechanisms into tumors. Breast Cancer Res. (2009) 11:S21. doi: 10.1186/bcr2282 20030873

[11] Chen N Li Y Ye Y Palmisano M Chopra R Zhou S. Pharmacokinetics and pharmacodynamics of nab-paclitaxel in patients with solid tumors: disposition kinetics and pharmacology distinct from solvent-based paclitaxel. J Clin Pharmacol. (2014) 54:1097–107. doi: 10.1002/jcph.304 PMC4302229 24719309

[12] Sun Q Bai X Sofias AM van der Meel R Ruiz-Hernandez E Storm G. Cancer nanomedicine meets immunotherapy: opportunities and challenges. Acta Pharmacologica Sin. (2020) 41:954–8. doi: 10.1038/s41401-020-0448-9 PMC7470866 32555445

[13] Bastaki S Irandous M Ahmadi A Hojjat-Farsangi M Ambrose P Hallaj S. PD-L1/PD-1 axis as a potent therapeutic target in breast cancer. Life Sci. (2020) 247:117437. doi: 10.1016/j.lfs.2020.117437 32070710

[14] Stovgaard ES Stovgaard ES Dyhl-Polk A Roslind A Balslev E Dorte N. PD-L1 expression in breast cancer: expression in subtypes and prognostic significance: a systematic review. Breast Cancer Res Treat. (2019) 174:571–84. doi: 10.1007/s10549-019-05130-1 30627961

[15] Shah M Osgood CL Amatya AK Fiero MH Pierce WF Nair A. FDA approval summary: pembrolizumab for neoadjuvant and adjuvant treatment of patients with high-risk early-stage triple-negative breast cancer. Clin Cancer Res. (2022) 28:5249–53. doi: 10.1158/1078-0432.CCR-22-1110 35925043

[16] Emens LA Middleton G. The interplay of immunotherapy and chemotherapy: harnessing potential synergies. Cancer Immunol Res. (2015) 3:436–43. doi: 10.1158/2326-6066.CIR-15-0064 PMC5012642 25941355

[17] Samanta D Park Y Ni X Semenza GL. Chemotherapy induces enrichment of CD47+/CD73+/PDL1+ immune evasive triple-negative breast cancer cells. Proc Natl Acad Sci. (2018) 115:E1239–48. doi: 10.1073/pnas.1718197115 PMC5819443 29367423

[18] Zhao P Xu Y Ji W Zhou S Li L Qiu L. Biomimetic black phosphorus quantum dots-based photothermal therapy combined with anti-PD-L1 treatment inhibits recurrence and metastasis in triple-negative breast cancer. J Nanobiotechnol. (2021) 19:1–18. doi: 10.1186/s12951-021-00932-2 PMC8201856 34120612

[19] Liu Y Qiu N Shen L Liu Q Zhang J Cheng YY. Nanocarrier-mediated immunogenic chemotherapy for triple negative breast cancer. J Controlled Release. (2020) 323:431–41. doi: 10.1016/j.jconrel.2020.04.040 PMC8601127 32360890

[20] Wilkerson AD Parthasarathy PB Stabellini N Mitchell C Pavicic PG Fu P. Phase II clinical trial of pembrolizumab and chemotherapy reveals distinct transcriptomic profiles by radiologic response in metastatic triple-negative breast cancer. Clin Cancer Res. (2024) 30:82–93. doi: 10.1158/1078-0432.CCR-23-1349 37882661 PMC10767305

[21] Emens LA Molinero L Loi S Rugo HS Schneeweiss A Dieras V. Atezolizumab and nab-paclitaxel in advanced triple-negative breast cancer: biomarker evaluation of the IMpassion130 study. J Natl Cancer Inst. (2021) 113:1005–16. doi: 10.1093/jnci/djab004 PMC8328980 33523233

[22] Røssevold AH Andresen NK Bjerre CA Gilje B Jakobsen EH Raj SX. Atezolizumab plus anthracycline-based chemotherapy in metastatic triple-negative breast cancer: the randomized, double-blind phase 2b ALICE trial. Nat Med. (2022) 28:2573–83. doi: 10.1038/s41591-022-02126-1 PMC9800277 36482103

[23] Wang Z Cordova LE Chalasani P Lu J. Camptothesome potentiates PD-L1 immune checkpoint blockade for improved metastatic triple-negative breast cancer immunochemotherapy. Mol Pharmaceutics. (2022) 19:4665–74. doi: 10.1021/acs.molpharmaceut.2c00701 PMC9744414 36413426

[24] Xu H Hu M Liu M An S Guan K Wang M. Nano-puerarin regulates tumor microenvironment and facilitates chemo- and immunotherapy in murine triple negative breast cancer model. Biomaterials. (2020) 235:119769. doi: 10.1016/j.biomaterials.2020.119769 31986348 PMC7093100

[25] Houthuijzen JM Jonkers J. Cancer-associated fibroblasts as key regulators of the breast cancer tumor microenvironment. Cancer Metastasis Rev. (2018) 37:577–97. doi: 10.1007/s10555-018-9768-3 30465162

[26] Feng B Niu Z Hou B Zhou L Li Y. Enhancing triple negative breast cancer immunotherapy by ICG-templated self-assembly of paclitaxel nanoparticles. Adv Funct Mater. (2020) 30:1906605. doi: 10.1002/adfm.201906605

[27] Lu Q Qi S Li P Yang L Yang S Wang Y. Photothermally activatable PDA immune nanomedicine combined with PD-L1 checkpoint blockade for antimetastatic cancer photoimmunotherapy. J Mater Chem B. (2019) 7:2499–511. doi: 10.1039/C9TB00089E 32255127

[28] Zou C Tang Y Zeng P Cui D Amili MA Chang Y. cRGD-modified nanoparticles of multi-bioactive agent conjugate with pH-sensitive linkers and PD-L1 antagonist for integrative collaborative treatment of breast cancer. Nanoscale Horizons. (2023) 8:870–86. doi: 10.1039/D2NH00590E 36987679

[29] Li Z Cai H Li Z Ren L Ma X Zhu H. A tumor cell membrane-coated self-amplified nanosystem as a nanovaccine to boost the therapeutic effect of anti-PD-L1 antibody. Bioactive Mater. (2023) 21:299–312. doi: 10.1016/j.bioactmat.2022.08.028 PMC9478499 36157245

[30] Zhang L Zheng H Cui H Yin X Zhang C Wang Y. Nanomicelle-based redox-responsive dual-prodrug for synergistic breast cancer chemo-immunotherapy. ACS Appl Nano Mater. (2023) 6:18263–74. doi: 10.1021/acsanm.3c03510

[31] Zhang R Zhang R Zhu Z Lv H Li F Sun S. Immune checkpoint blockade mediated by a small-molecule nanoinhibitor targeting the PD-1/PD-L1 pathway synergizes with photodynamic therapy to elicit antitumor immunity and antimetastatic effects on breast cancer. Small. (2019) 15:1903881. doi: 10.1002/smll.201903881 31702880

[32] Cha J-H Yang WH Xia W Wei Y Chan L Lim S. Metformin promotes antitumor immunity via endoplasmic-reticulum-associated degradation of PD-L1. Mol Cell. (2018) 71:606–620. e7. doi: 10.1016/j.molcel.2018.07.030 30118680 PMC6786495

[33] Hu C He X Chen Y Yang X Qin L Lei T. Metformin mediated PD-L1 downregulation in combination with photodynamic-immunotherapy for treatment of breast cancer. Adv Funct Mater. (2021) 31:2007149. doi: 10.1002/adfm.202007149

[34] Cai S Chen Z Wang Y Wang M Wu J Tong Y. Reducing PD-L1 expression with a self-assembled nanodrug: an alternative to PD-L1 antibody for enhanced chemo-immunotherapy. Theranostics. (2021) 11:1970. doi: 10.7150/thno.45777 33408792 PMC7778587

[35] Zhu H Ma K Ruan R Yang C Yan A Li J. Tumor-targeted self-assembled micelles reducing PD-L1 expression combined with ICIs to enhance chemo-immunotherapy of TNBC. Chin Chem Lett. (2024) 35:108536. doi: 10.1016/j.cclet.2023.108536

[36] Zhang R Cheng G Liu S Lv H Li J. A four-in-one pure nanomedicine for synergistic multi-target therapy against breast cancer. J Mater Chem B. (2021) 9:8809–22. doi: 10.1039/D1TB01820E 34633023

[37] Lang T Zheng Z Huang X Liu Y Zhai Y Zhang P. Ternary regulation of tumor microenvironment by heparanase-sensitive micelle-loaded monocytes improves chemo-immunotherapy of metastatic breast cancer. Adv Funct Mater. (2021) 31:2007402. doi: 10.1002/adfm.202007402

[38] Pham LM Poudel K Ou W Phung CD Nguyen HT Nguymen BL. Combination chemotherapeutic and immune-therapeutic anticancer approach via anti-PD-L1 antibody conjugated albumin nanoparticles. Int J Pharmaceutics. (2021) 605:120816. doi: 10.1016/j.ijpharm.2021.120816 34161810

[39] Cheng Y Wang C Wang H Zhang Z Yang X Dong Y. Combination of an autophagy inhibitor with immunoadjuvants and an anti-PD-L1 antibody in multifunctional nanoparticles for enhanced breast cancer immunotherapy. BMC Med. (2022) 20:411. doi: 10.1186/s12916-022-02614-8 36303207 PMC9615197

[40] Zhang P Qin C Liu N Zhou X Chu X Lv F. The programmed site-specific delivery of LY3200882 and PD-L1 siRNA boosts immunotherapy for triple-negative breast cancer by remodeling tumor microenvironment. Biomaterials. (2022) 284:121518. doi: 10.1016/j.biomaterials.2022.121518 35462305

[41] Bahreyni A Qin C Liu N Zhou X Chu X Lv F. Engineering a facile and versatile nanoplatform to facilitate the delivery of multiple agents for targeted breast cancer chemo-immunotherapy. Biomed Pharmacother. (2023) 163:114789. doi: 10.1016/j.biopha.2023.114789 37119737

[42] Yuan Y Cai T Xia X Zhang R Chiba P Cai Y. Nanoparticle delivery of anticancer drugs overcomes multidrug resistance in breast cancer. Drug Deliv. (2016) 23:3350–7. doi: 10.1080/10717544.2016.1178825 27098896

[43] Desai N. Challenges in development of nanoparticle-based therapeutics. AAPS J. (2012) 14:282–95. doi: 10.1208/s12248-012-9339-4 PMC3326161 22407288

[44] Gavas S Quazi S Karpiński TM. Nanoparticles for cancer therapy: current progress and challenges. Nanoscale Res Lett. (2021) 16:173. doi: 10.1186/s11671-021-03628-6 34866166 PMC8645667

[45] Shi J Kantoff PW Wooster R Farokhzad OC. Cancer nanomedicine: progress, challenges and opportunities. Nat Rev Cancer. (2017) 17:20–37. doi: 10.1038/nrc.2016.108 27834398 PMC5575742

