# Supplementary material for: Nanoparticle-enhanced PD-1/PD-L1 targeted combination therapy for triple negative breast cancer
Source: Front Oncol. 2024 May 2;14:1393492. doi: 10.3389/fonc.2024.1393492 (PMC11096478; doi:10.3389/fonc.2024.1393492)
Supplement: Supplementary file 1 [file Table_1.docx]

Supplementary Material

# Table S1. Examples of recent nanoparticle strategies for enhanced PD-1/PD-L1 inhibitor-based combination therapy for TNBC.

| **NP type** | **NP preparation method** | **Therapeutic agents in NP** | **Therapeutic efficacy** | **Safety/toxicity** | **Ref. #** |
| --- | --- | --- | --- | --- | --- |
| Liposome | Thin film hydration method made with Lipo-Sphingomyelin/Cholesterol | - Camptothecin: induce ICD, upregulate PD-L1, cytotoxicity | - Upregulated PD-L1 levels 2.5 × - With αPD-L1, TIR*: 80-90% - Eradicated lung metastasis | No significant mouse weight loss in all treatment groups | (23) |
| Liposome | Thin film dispersion method made with DSPE-PEG-AEAA, DSPE-PEG, and DOPC | - 17-AAG: Hsp90 inhibitor | - Increased tumor infiltrating T cell population by 10× - Increased memory CD8+ T cells to 7% - With αPD-L1, tumor mass reduced by half - Eradicated lung metastasis | - No morphological differences observed in major organs between treatment groups - No severe hepatic or renal malfunction between treatment groups - No significant body weight loss occurred throughout treatments | (19) |
| Cancer Cell Membrane Coated Quantum Dots | - Black phosphorous quantum dots were prepared by sonication assisted liquid phase exfoliation - Then mixed with cancer cell membranes and resuspended in PBS | - Black phosphorous: cytotoxicity, induce ICD w/ NIR | - Increased dendritic cell population by 3.29x - With αPD-L1, nearly eradicated primary tumor - Eradicated lung metastasis - 100% of the mice survived after 60 days | - Body weight growth was not changed in all treatment groups | (18) |
| Nano emulsion | Used lecithin as an emulsifier to carry puerarin and modified NP with AEAA | - Puerarin: deactive TAFs | - Reduced TAFs to 3.5% of total cells - Reduced collagen deposition to 2.6% of total cells - Reduced tumor mass by half - Median survival time was 56 days | - No hepatic or renal dysfunctions in PBS, blank emulsion, and NanoPue groups - No inflammation or necrosis found in heart, liver, spleen and kidney of mice treated with blank emulsion or Nano Pue - No significant weight loss occurred for all mice | (24) |
| Self-Assembled | Paclitaxel in DMSO was mixed with Indocyanine Green in aqueous solution, purified by centrifugation and ultrafiltration which was re-dispersed in DI water | - Indocyanine Green: induce ICD, cytotoxicity w/NIR - Paclitaxel: induce ICD, cytotoxicity, kill Tregs | - Increased dendritic cell maturation to 50.1% - Reduced Tregs by 10-15% - With αPD-L1, nearly eradicated primary tumor - Prevented lung metastasis - 75% of the mice survived after 45 days | - Body weight of mice remained unaffected by treatment with ISPN +NIR + αPD-L1 - No histopathological damage of the major organs were found in the ISPN +NIR +αPD-L1 | (26) |
| Polymeric | - Dopamine hydrochloride was added to ammonia and ethanol aqueous solution and washed with DI water - mPEG-NH2 was added, the solution was washed - Carbon dots and R848 were added to the solution | - Carbon Dots: induce ICD, cytotoxicity w/NIR - R848: release antigens | - 2.1% cytotoxic T lymphocyte population - With αPD-L1, TIR: 75% - With αPD-L1, completely inhibited growth of distant tumors - 80% of mice survived after 30 days | - No significant effects on major organs with treatment of PDA-PEG-R848 | (27) |
| cRGD Drug Conjugate | D-α-tocopherol polyethylene glycol 1000 succinate was synthesized to TPA, then conjugated with Doxorubicin. Adjudin was then added to the solution. | - Doxorubicin: cytotoxicity, induce ICD - Adjudin: anti-metastasis - TPGS: ROS inducer | - Increased ROS levels 2.3× - With αPD-L1, TIR of 84.08% - Prevented lung metastasis | - No difference shown in blood biochemical and histological analysis between saline group and cRGD-TDA NPs - cRGD-TDA NPs showed less toxicity than free doxorubicin by itself and combined with free adjudin according to blood biochemical analysis - No obvious damage to major organs | (28) |
| Zeolitic Imidazolate Framework (ZIF-8) | - Epirubicin, Gox, and hemin were mixed with 2-methylimidazole solution, stirred and centrifuged to obtain precipitates - 4T1 Cell membrane solution was added to nanoparticle solution | - Epirubicin: induce ICD, cytotoxicity - Glucose Oxidase: ROS inducer - Hemin: ROS inducer | - 10× more epirubicin was released in pH 5.4 than pH 7.4 - Increased ROS by 2.5x - With αPD-L1, 82.02% TIR - Prevented lung metastasis | - No significant body weight change in mice treated with mEHGZ - No obvious pathologic changes in major organs in mice treated with mEHGZ + anti-PD-L1 antibody | (29) |
| Polymeric Nanocarrier | - The block copolymer PEG-PLys was synthesized by ring-opening polymerization of Lys(TFA)-NCA, initiated by mPEG-NH2, and subsequently purified. - CPT-ss-OH and JQ1-COOH prodrugs were synthesized separately, involving reactions with triphosgene and respective substrates, followed by purification. - These prodrugs were then conjugated to PEG-PLys to form PEG-PLys(ss-CPT) and PEG-PLys(ss-JQ1) through controlled chemical reactions and subsequent purification steps. | - Camptothecin: cytotoxicity, induce ICD - JQI: block PD-L1 | - TIR: 80.3% | - Hemolysis Test: The hemolysis rate was low, indicating minimal red blood cell disruption. - Histological Analysis: Major organs (heart, liver, spleen, lung, and kidney) from mice treated with the nanomedicine showed no evidence of severe tissue damage or inflammation in histological sections | (30) |
| Self-assembled w/ Reprecipitation | Synthesized using reprecipitation, mixing BMS-202 in acetone (good solvent) with large volume of water (poor solvent) | - BMS 202: block PD-L1 - Ce6: induce ICD, cytotoxicity w/NIR | - 91.1% TIR | - No significant histological damage was observed in major organs - No significant changes in body weight | (31) |
| Metformin Conjugated to Ce6 through MMP-2 cleavable peptide | - Metformin-1,4-phthalaldehyde molecules were synthesized and then conjugated to a GPLGVRGDK-Dde peptide. - Chlorin e6 was attached to the peptide to form MA-pepA-Ce6 via an amide linkage. - Nanoparticles were produced by ultrasonic emulsification of the compound in a solvent mixture, followed by dialysis to remove solvents and purification by centrifugation. | - Metformin: degrade PD-L1 - Ce6: induce ICD and cytotoxicity w/NIR | - Reduced tumor weights by 61.5%. - 50% reduction in PD-L1 expression on 4T1 tumor cells - Decrease in metastatic nodules in the lungs | - No significant tissue damage observed in major organs - No noticeable changes in body weight across treatment groups. - Maintained general health, indicating high biocompatibility | (33) |
| Self-assembled | Metformin and SN38 solutions individually added dropwise to double distilled water | - Metformin: degrade PD-L1 - SN38: induce ICD, cytotoxicity | - Reduced PD-L1 expression 4× - 80% TIR - Prevented lung metastasis - 50% survival rate after 30 days | - No significant abnormalities or lesions in major organs - Stable body weight across all treatment groups - Normal results in routine blood assays and chemistry, indicating no systemic toxicity | (34) |
| Micelles | - PEGCG and MET self-assembled into micelles via hydrogen bonds and electrostatic interactions to form PM micelles. - DOX was encapsulated into these PM micelles | - PEGCG: immune modulator - Metformin: block PD-L1 - Doxorubicin: cytotoxicity, increase ICD | - Reduced MDSCs by 10% - With anti-PD-1, 68.8% TIR | - No significant damage in major organs based on histological analysis - Stable body weight throughout the treatment - Normal liver function markers, indicating no liver toxicity | (35) |
| Self-assembled | Repertaxin, paclitaxel, BMS-1, Combretastatin in tetrahydrofuran was added into distilled water under vigorous stirring at room temperature, then tetrahydrofuran was removed by dialysis | - Paclitaxel: induce ICD - Repertaxin: kills cancer stem cells - BMS-1: block PD-L1 - Combretastatin: cut off blood supply in tumor microenvironment | - Reduced cancer stem cells to 4.85% - 92.5% TIR - Lung metastasis suppression > 90% - 50% of the mice survived after 95 days | - No significant weight loss of mice. Systemic toxicity may be mainly caused by paclitaxel. - No damage in major organs of tumor-bearing mice. | (36) |
| Micelles | - Heparan sulfate was conjugated to docetaxel and NLG919 - These compounds could self-assemble into micelles through film dispersion method | - Docetaxel: induce ICD, cytotoxicity - NLG919: inhibit indoleamine 2,3 dioxygenase - HY19991: inhibit PD-L1 | - 6.85, 3.96, 3.11 times higher intratumoral drug concentrations for the 3 drugs when carried by monocyte - TIR over 90% - Suppressed lung metastasis by 98.20% - 66.67% of the mice survived after 60 days | No severe body weight loss. Docetaxel caused elevated alanine aminotransferase and aspartate aminotransferase levels, and decreased white blood cells and granulocytes numbers. | (37) |
| Human Serum Albumin | - PTX was dissolved in 9:1 chloroform:ethanol and HSA was dissolved in water. - The organic and aqueous phases were homogenized. The dispersion was evaporated. - The PTX@HSA was centrifuged, and then the polymer PD-L1-PLL was coated on surface by electrostatic interaction. | - Paclitaxel: cytotoxicity, induce ICD - Anti-PD-L1 antibody: block PD-L1 | Combined with anti-CTLA-4 antibody, decreased tumor mass by 80% | No significant body weight change in mice. No anomalies in any of the main organs of the mice. | (38) |
| Polymeric | - Thin film hydration of PEI-OA, Soluplus and Solutol - Added to CpG and Ovalbumin solution - Added to an atezolizumab solution | - Paclitaxel: cytotoxicity, induce ICD - Chloroquine: autophagy inhibitor - Ovalbumin: antigen - CpG: immuno-potentiator - anti-PD-L1 antibody: block PD-L1 | - Autophagosome formation increased 4× - TIR: 80% - 60% of the mice survived after 60 days | No significant body weight change in mice. | (39) |
| Lipid + Protamine Inner Core | Nanocore is assembled through charging interaction between protamine and siPD-L1. Pro-siPD-L1 is mixed with LY loaded MMP2-responsive liposomes and co-extruded to prepare the nanosystem. | - LY3200882: inhibits TAF - PD-L1 siRNA: block PD-L1 | - Down-regulated alpha-SMA by 31.5% in NIH/3T3 cells - Down-regulated Collagen 1 by 52.4% in NIH/3T3 cells - Decreased tumor mass ~85% | - No significant body weight change of mice. - No non-specific uptake by non-tumor tissues. | (40) |
| Hyaluronic Acid Complex | - DsiRNA was added to Melittin to form Mel/PD-L1 DsiRNA - Doxrubicin was added to Mel/PD-L1 DsiRNA solution - Then human albumin was added to DoxMel/PD-L1 DsiRNA solution - The final product is collected by centrifuge. | - PD-L1 DsiRNA: inhibits PD-L1 expression - Doxorubicin: induce ICD, cytotoxicity - Melittin: activate MMP-2, cytotoxicity, immuno-adjuvant | - 3-fold higher uptake of drugs with HA - 75% decrease in PD-L1 expression - Decreased tumor weight by about 75-80% - 100% survival rate after 40 days | No significant body weight change of mice. | 41 |

*TIR=Tumor Inhibition Rate
